# Angiogenesis and evading immune destruction are the main related transcriptomic characteristics to the invasive process of oral tongue cancer

**DOI:** 10.1038/s41598-017-19010-5

**Published:** 2018-01-31

**Authors:** Juan Alberto Pérez-Valencia, Francisco Prosdocimi, Italo M. Cesari, Igor Rodrigues da Costa, Carolina Furtado, Michelle Agostini, Franklin David Rumjanek

**Affiliations:** 1 Instituto de Bioquímica Médica Leopoldo de Meis, Centro de Ciências da Saúde, Universidade Federal do Rio de Janeiro, Rio de Janeiro, RJ Brazil; 2 Instituto Nacional do Câncer (INCA), Rio de Janeiro, RJ Brazil; 3 Departamento de Patologia e Diagnóstico Oral, Faculdade de Odontologia, Universidade Federal do Rio de Janeiro, Rio de Janeiro, RJ Brazil

## Abstract

Metastasis of head and neck tumors is responsible for a high mortality rate. Understanding its biochemistry may allow insights into tumorigenesis. To that end we carried out RNA-Seq analyses of 5 SCC9 derived oral cancer cell lines displaying increased invasive potential. Differentially expressed genes (DEGs) were annotated based on *p-values* and false discovery rate (*q-values*). All 292 KEGG pathways related to the human genome were compared in order to pinpoint the absolute and relative contributions to the invasive process considering the 8 hallmarks of cancer plus 2 new defined categories, as well as we made with our transcriptomic data. In terms of absolute contribution, the highest correlations were associated to the categories of evading immune destruction and energy metabolism and for relative contributions, angiogenesis and evading immune destruction. DEGs were distributed into each one of all possible modes of regulation, regarding up, down and continuum expression, along the 3 stages of metastatic progression. For *p-*values twenty-six genes were consistently present along the tumoral progression and 4 for *q-*values. Among the DEGs, we found 2 novel potentially informative metastatic markers: PIGG and SLC8B1. Furthermore, interactome analysis showed that MYH14, ANGPTL4, PPARD and ENPP1 are amenable to pharmacological interventions.

## Introduction

Head and Neck Squamous Cell Carcinoma (HNSCC) is the sixth most common cancer worldwide, with more than 600000 new cases per year^1^. Among these, the oral tongue squamous cell carcinoma (OTSCC) is the most prevalent cancer, with high incidence of metastasis to the lymph nodes of the neck^2,3^ being responsible for a decrease in the overall survival rates by nearly 50%^4–6^.

Even though considerable research efforts, so far there is as yet no clear consensus about the genetic alterations that underlie nodal metastasis and the metastatic process itself.

As a strategy to identify major patterns of expression, next generation sequencing (NGS) has been used to explore not only the genetic heterogeneity and gene expression of diverse types of cancer, but also those aspects related to tumor progression. In the present work we have examined the applicability of high throughput gene expression analyses as a resource to investigate major alterations in expression patterns of tumor cells as they increasingly acquire a metastatic phenotype. Besides, such an approach can reveal novel biomarkers of OTSCC.

To that end, we used as study model SCC-9 primary tumor (ATCC CRL-1629) as well as 4 cell lines derived previously established by Agostini and collaborators^7^: ZsG, SCC9-transduced with a green fluorescent protein; and 3 metastatic cell generations carrying the fluorescent protein. The cells displaying increasing invasive properties were referred to as LN1, LN2 and LN3^7,8^.

Frequently, the differentially expressed genes (DEGs) are selected by either *t*-test or post-test corrections, and subsequent analyses are made based on one of these selections. However, the quality of the results depends on the number of genes analyzed, based on cut-offs of fold change and statistical significance^9^. In the present study, we compared the impact of the standard statistics cut-offs on the analyses. In this manner we initially identified the DEGs using the student’s *t*-test (*p*-values) and its false discovery rate (FDR) correction (*q*-values). Then, all DEGs were divided according to the standard *p*- and *q*-values, setting the cut-off α = 0.05. DEGs were sorted as protein coding genes (PCG) and non-coding genes (NCG). Using the PCG, we obtained the related KEGG pathways and classified them according to the 8 consensual hallmarks of cancer: (i) auto-sustained proliferative signaling, (ii) ability to evade growth suppressors, (iii) mechanisms to resist cell death, (iv) enabling of replicative immortality, (v) angiogenesis induction, (vi) invasion and metastasis capacity, (vii) shift of energy metabolism, and (viii) evasion of immune destruction^10^. We found 71 KEGG pathways related to other cancer types and chronic diseases. Based on the assumption that individual genes may take part in more than one pathway, the approach involving the hallmarks allowed us acknowledged alternative functions for each gene considered.

Furthermore, in undertaking to extend the observations pertaining to the individual contributions (absolute and relative) to invasion and metastasis, of genes sorted according to the hallmarks of cancer and including the two extra classes proposed by us, we matched the KEGG pathways associated to each hallmark against the 77 KEGG pathways of the invasion and metastasis category.

The results indicated that the PCGs of DEGs involved in angiogenesis and immune destruction evasion displayed the highest contribution to metastasis in OTSCC for both *p*- and *q*-values data. In contrast, PCGs related to energy metabolism and other cancer types represented less the relative contributions to invasiveness. Energy metabolism was the second hallmark with highest number of shared genes with invasion and metastasis, while evading immune destruction was the first. The hallmark “other cancer types” had the lowest contributions to progression towards metastasis for both *p*- and *q-*values data. The results highlighted a strong correlation between the data analyses of uncorrected (Student’s *t*–test [*p*-values]) or corrected (FDR [*q-*values]) and their patterns of contributions to invasiveness.

The comparative analyses were applied to all the 11 types of gene regulation that DEGs possibly display, referred as clusters of gene expression (CoGE): (i) exclusive to parental cell line, (ii) exclusive to derived cell line, (iii) continuum (similar expression values), (iv) exclusive down regulated in parental cell line, (v) exclusive up regulated in parental cell line, (vi) exclusive down regulated in derived cell line, (vii) exclusive up regulated in derived cell line, (viii) common down regulated, (ix) common up regulated, (x) common down regulated in parental and up regulated in derived cell lines and (xi) common up regulated in parental and down regulated in derived cell lines. By comparing those clusters, we found 26 DEGs sequentially altered in the OTSCC model. Of these, 15 were down regulated; 1 was up regulated and 10 displayed only slight modifications of expression (continuum). Also, CoGE analyses showed differences between proliferation, metabolism and the mechanisms related to promotion of the metastatic process. This set of 26 genes may constitute biomarkers of OTSCC metastasis.

## Results

The total amount of partial RNA sequences exceeded 100 million reads with 50–250 bp for each lineage. The experimental design, number of reads and number of human genes mapped are shown in Supplementary Table 1.

### Tongue cancer differentially expressed genes represent almost all of the reported human KEGG pathways

In order to understand main changes on gene expression along with the metastatic OTSCC progression we used the software Cufflinks to map the reads of the known human genes. The relative abundance metric parameter FPKM (Fragments Per Kilobase of exon per Million reads sequenced) was used to represent the value of gene expression on each dataset analyzed. To detect DEGs, we applied the Student’s *t*-test (*p*-values), and then, the FDR correction (*q*-values). The quality of the results depends on the amount of genes analyzed, which in turn is based on cut-offs and statistical significance^9^. This justified the use of both data, *p-* and *q-*values. We compared the expression of genes between the parental *versus* its derived cell line. Regarding the *p*-value, 9169 DEGs were found between SCC9 *vs*. ZsG; 11597 between ZsG *vs*. LN1, 5011 between LN1 *vs*. LN2, and 8572 between LN2 *vs*. LN3. Regarding the *q*-values, 6728 DEGs were found between SCC9 *vs*. ZsG; 9874 between ZsG *vs*. LN1, 284 between LN1 *vs*. LN2 and 5579 between LN2 *vs*. LN3 (Supplementary Table 2). Hereafter, only the identified DEGs will be discussed.

**Figure 1:**
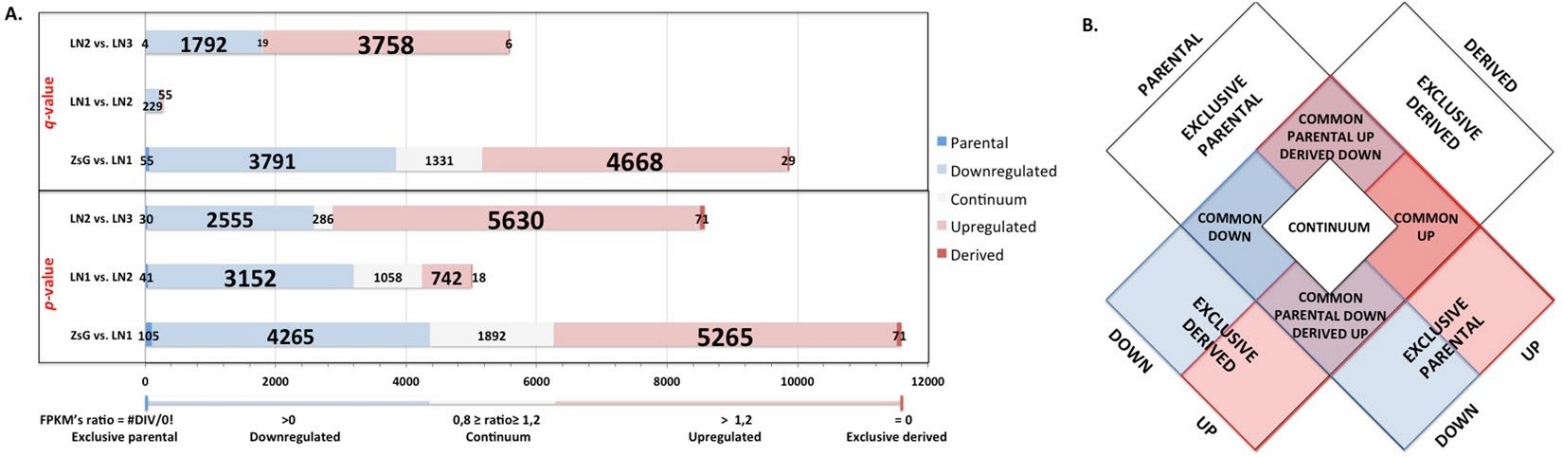
Regulation of the differentially expressed genes. (**A**) Exclusive, down regulated, continuum and up regulated for *p-* and *q-* values. (**B**) Clusters of gene expression after comparison of the regulated genes from parental and its derived cell lines. In white, DEGs without regulation of expression (exclusive to parental or to derived cell lines and continuum), in blue, down regulated DEGs and in red, up regulated DEGs; light purple, intersections between down regulated and up regulates DEGs.

Expression ratios were obtained between parental and derived cell lines. Based on that, a ranking of all DEGs was produced (Supplementary Table 3). SCC9 cell line was used as the reference for ZsG transformed cells, however the following comparisons of transformed cell lines (TCLs) (ZsG *vs*. LN1, LN1 *vs*. LN2 and LN2 *vs*. LN3) will be discussed. Down and up regulated genes were identified for each comparison (Fig. 1A), and by matching those differentially expressed genes, we could classify them into 11-different clusters of gene expression (CoGE). These strategy allowed the identification of regulatory patterns among parental-derived changes on gene expression: (i) exclusive parental genes (FPKM > 0 in parental cell line and FPKM = 0 in derived cell line); (ii) exclusive derived genes (FPKM > 0 in derived cell line and FPKM = 0 in parental cell line); (iii) continuum (referring to those genes whose expression did not change significantly between parental and derived cell line displaying FPKM ratio 0.8 ≥ x ≥ 1.2. Differences of 0.1 are common even in technical replicates, but differences of 0.3 are considered as significant variations. With that in mind, the value of 1 ± 0.2 was set as the limit for the continuum cluster. A similar approach was used for human neoplasms^11^); (iv) exclusive parental down regulated (FPKM ratio < 0.8); (v) exclusive parental up regulated (FPKM ratio > 1,2); (vi) exclusive derived down regulated (FPKM ratio < 0.8); (vii) exclusive derived up regulated (FPKM ratio > 1.2); (viii) common down regulated; (ix) common up regulated; (x) common parental down regulated and derived up regulated; and (xi) common parental up regulated and derived down regulated (Table 1 and Fig. 1B).

**Table 1:** Number of protein coding genes (PCG), KEGG pathways (Path) and non-coding genes (NCG) differentially expressed for each cluster of gene expression between parent-derived cell lines of tongue metastatic progression, for *p-* and *q-*values.

Differentially expressed genes	*p*-values									*q*-values								
	ZsG vs. LN1			LN1 vs. LN2			LN2 vs. LN3			ZsG vs. LN1			LN1 vs. LN2			LN2 vs. LN3		
	PCG	Path	NCG	PCG	Path	NCG	PCG	Path	NCG	PCG	Path	NCG	PCG	Path	NCG	PCG	Path	NCG
Exclusive parental	16	20	45	3	9	28	3	5	16	7	10	23	0	0	0	1	2	3
Exclusive derived	7	12	46	7	15	8	9	12	46	5	11	16	0	0	0	1	1	4
Continuum	665	252	51	401	251	23	98	191	6	466	241	23	0	0	0	6	10	0
Exclusive parental DW	236	229	191	999	284	767	340	226	171	213	227	116	1108	284	597	34	98	5
Exclusive parental UP	273	233	199	1028	275	541	68	136	121	245	219	100	1337	278	462	3	12	0
Exclusive derived DW	479	268	504	400	236	163	552	266	285	477	260	399	17	50	1	521	259	167
Exclusive derived UP	757	257	420	71	130	116	1130	278	759	751	260	335	3	7	0	1119	277	474
Common DW	70	130	19	148	179	43	146	190	18	60	123	11	16	27	2	25	72	3
Common UP	41	123	13	57	129	16	29	73	8	26	109	8	11	50	1	3	6	0
Common parental DW derived UP	724	265	242	45	92	54	494	247	142	619	261	127	4	11	1	22	53	2
Common parental UP derived DW	645	268	341	434	242	123	76	139	57	596	267	190	48	124	7	12	45	2

Using STRING^12^, we further categorized the DEGs for both *p*-values and *q*-values into protein coding genes (PCG) (Supplementary Tables 4A and 4B, *p-* and *q*-values, respectively) and non-coding genes (NCG), (Supplementary Tables 5A and 5B, *p-* and *q*-values, respectively). Also, STRING allowed the enrichment of the interactome of all differentially expressed PCG with KEGG pathways by assigning the molecular or biochemical pathways related to them (Table 1 and Supplementary Table 6). Supplementary Tables 6A (*p-* values) and 6B (*q-* values) show the KEGG pathways and genes related to each hallmark of cancer, related to each CoGE.

Table 1 reveals that there was no correlation between the number of PCG and KEGG pathways, for both *p-* and *q-*values. Concerning the KEGG pathways, the highest was 284 (corresponding to LN1 vs. LN2, exclusive parental down regulated, for both *p-* and *q-*values). This observation raised the question of how many KEGG pathways are related to the cDNA of the human genome. Using all the 35238 annotated genes, we followed the same procedures, obtaining 292 KEGG pathways related to 7858 PCG (Supplementary Table 6C). With this approach almost all the pathways related to the human genome were included in our analysis. However, two of these were not detected: D-arginine and D-ornithine metabolism, and fatty acid elongation in mitochondria. The same strategy was applied to all 3717 DEGs found in our analyses of *q-*values (Supplementary Table 6D).

### KEGG pathways and protein-coding genes related to ‘Energetic metabolism’ and ‘Invasion and metastasis’ hallmarks were the most represented

All 292 KEGG pathways of the human genome were distributed according to the 8 hallmarks of cancer: (i) auto-sustained proliferative signaling, (ii) ability to evade growth suppressors, (iii) mechanisms to resist cell death, (iv) enable replicative immortality, (v) angiogenesis induction, (vi) invasion and metastasis capacity, (vii) shift of energy metabolism and (viii) evasion of immune destruction^10^, using the PubMed database by manual curation. Because (ix) other cancer types and (x) chronic diseases we related to 71 KEGG pathways, these were added to the 8 hallmarks of cancer. Energy metabolism was the hallmark with the highest number of KEGG pathways (123), followed by invasion and metastasis (77), chronic diseases (52), proliferative signaling (39), resisting cell death and evading immune destruction (37), angiogenesis (24), evading growth suppressors (22), other cancer types (21) and replicative immortality (18).

**Figure 2:**
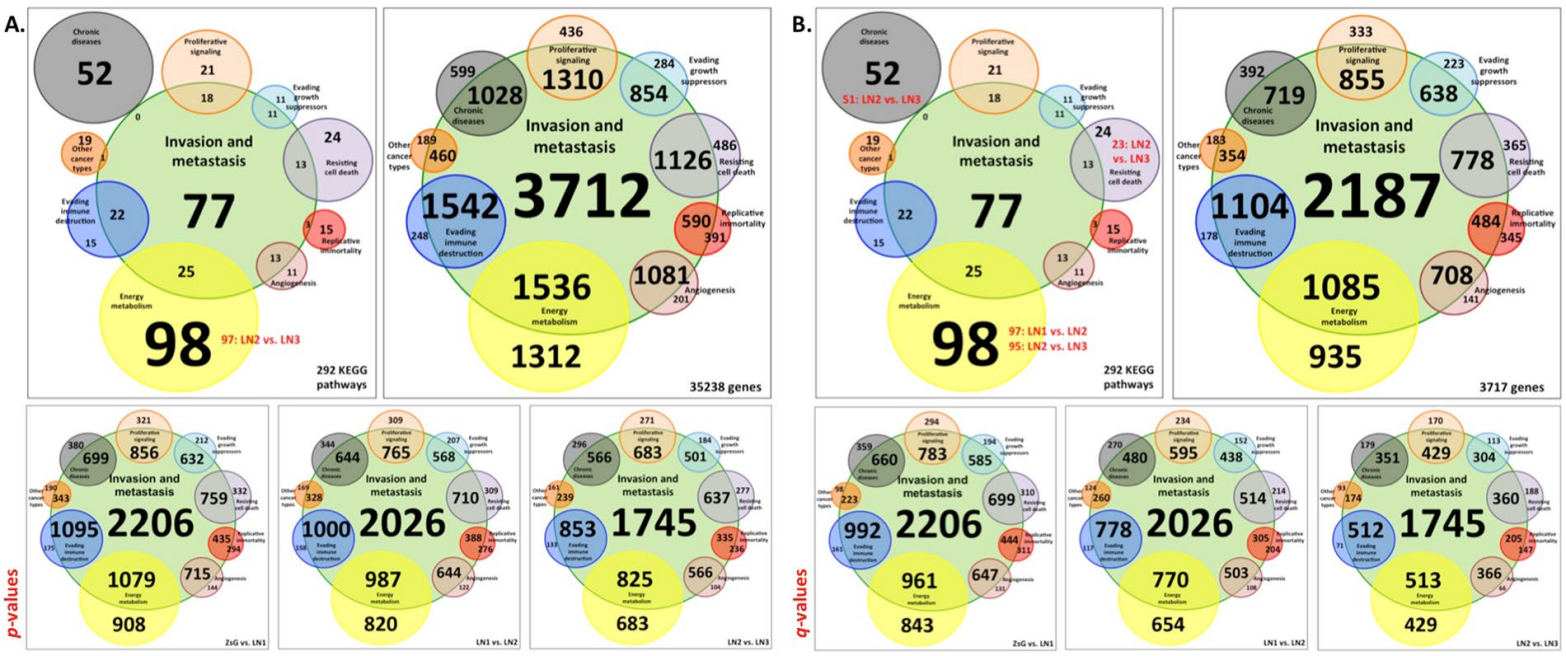
Contributions of each hallmark of cancer to invasion and metastasis, based on the KEGG pathways from protein coding genes. The intersections show the number of KEGG pathways (upper left panels) or genes (upper right panels). (**A**) All 292 KEGG pathways of the human genome (upper left panel) or genes related to 35238 human cDNAs annotated in Ensemble (upper right panel) and the distribution of the differentially expressed genes in all-3 comparisons of transformed cell lines (bottom panels, ZsG *vs*. LN1 left, LN1 *vs*. LN2 middle and LN2 *vs*. LN3 right), for *p-*values; (**B**) all 292 KEGG pathways of the human genome (upper left panel) or related to 3717 differentially expressed genes after FDR correction found in this study (upper right panel) and the distribution of the differentially expressed genes in all 3 comparisons of transformed cell lines (bottom panels, ZsG *vs*. LN1 left, LN1 *vs*. LN2 middle and LN2 *vs*. LN3 right) for *q-*values. In upper left panels were highlighted (in red) the changes of the number of KEGG pathways found in the transformed cell lines comparisons.

The characteristic chosen to compare all other hallmarks was invasion and metastasis. Of the 77 KEGG pathways related to invasion and metastasis, 25 were shared with energy metabolism, 22 with immune destruction evasion, 18 with proliferative signaling, 13 with cell death resistance and angiogenesis, 11 with growth suppression evasion and 3 with replicative immortality. Also, other cancer types and chronic diseases pathways were compared to those belonging to the invasion and metastasis hallmark. We found 1 and 0 shared pathways respectively, as depicted in Fig. 2A, upper left panel.

When the same comparison was carried out for *p-*values, a similar distribution was found except for an exclusive pathway of energy metabolism: 97 out of 98, when LN2 and LN3 were compared (Fig. 2A, upper panel, in red). For *q*-values, 23 of 24 exclusive pathways were assigned to the category of resistance to cell death when LN2 and LN3 were compared. There was a decrease in energy metabolism exclusive pathways, for the comparisons LN1 *vs*. LN2 (97 out of 98) and LN2 *vs*. LN3 (95 out of 98); and 51 out of 52 chronic diseases pathways when LN2 *vs*. LN3 were compared (Fig. 2B, upper panel, in red).

In order to evaluate the absolute contributions of the genes associated to invasion and metastasis, the same approach was followed, using the related DEGs of each KEGG pathway identified by STRING. First, we compared all 35238 human genes against invasion and metastasis. The highest number of PCG was related to invasion and metastasis (3712) in which 1542 were shared with evasion of immune destruction followed by 1536 with energy metabolism, 1310 with proliferative signaling, 1126 with resistance to cell death, 1081 with angiogenesis, 1028 with chronic diseases, 854 with evading growth suppressors, 590 with replicative immortality and 460 with other cancer types, with which it shared only one KEGG pathway (Fig. 2A, upper right panel).

We used the same approach for *p-*values, finding for ZsG *vs*. LN1, 2206 DEGs related to invasion and metastasis. For LN1 *vs*. LN2, there were 2026 DEGs and for LN2 *vs*. LN3, 1745 DEGs (Fig. 2A, bottom panels right, middle and left, respectively). All 3 TCLs comparisons displayed the same pattern of contributions to invasion and metastasis.

The same analyses were carried out for *q*-values. For that we used all 3717 DEGs found in our outcomes. The results are shown in Fig. 2B. The left upper panel displays the DEGs related to KEGG pathways and the upper right panel displays the DEGs associated to each hallmark of cancer. Of those, 2187 DEGs were related to invasion and metastasis. The related DEGs to invasion and metastasis are shown in Fig. 2B, where ZsG *vs*. LN1 displayed 1977, LN1 *vs*. LN2 1.511, and LN2 *vs*. LN3, 1030 (bottom panels, right, middle and left, respectively).

### ‘Angiogenesis’ and ‘Evading immune destruction’ are the main hallmarks related to metastasis

Next we enquired the relative DEGs contributions of the hallmarks to invasion and metastasis. Accordingly, we compared all DEGs of each hallmark against those of invasion and metastasis, and looked for gene redundancy. This was carried out by matching each of the 11-CoGE to each of the 8 + 2 hallmarks of cancer. Panels 1 and 3 depicted in Table 2 (*p*- and *q*-values, respectively) contain the gene number of each CoGE related to each 8 + 2 hallmarks. See also Supplementary Tables 7A (*p*-values) and 7B (*q*-values), which show the KEGG pathways and their related gene IDs for each CoGE. In addition, panels 2 and 4 of Table 2 show the percentage contributions of each hallmark to invasion and metastasis, distributed into each CoGE. This information allowed us to recognize which hallmark was the closest to the invasive process. The number of genes for each hallmark is described in section 2.

**Table 2:** Number of differentially expressed genes (DEGs) for each comparison between the transformed cell lines in 11-clusters of gene expression into the 8 + 2 hallmarks of cancer (panels 1 and 3) and percentages of the genetic contribution of each hallmark of cancer to the invasion and metastasis process (panels 2 and 4).

	Clusters of gene expression	*p*-values						*q*-values					
		# of genes			% of contribution			# of genes			% of contribution		
		ZsG vs. LN1	LN1 vs. LN2	LN2 vs. LN3	ZsG vs. LN1	LN1 vs. LN2	LN2 vs. LN3	ZsG vs. LN1	LN1 vs. LN2	LN2 vs. LN3	ZsG vs. LN1	LN1 vs. LN2	LN2 vs. LN3
**Invasion and metastasis**	Exclusive Parental	9	3	1	100,0	100,0	100,0	4	0	0	100,0	100,0	100,0
	Exclusive Derived	3	5	6	100,0	100,0	100,0	3	0	0	100,0	100,0	100,0
	Continuum	381	252	60	100,0	100,0	100,0	269	0	2	100,0	100,0	100,0
	Exclusive Parental DW	144	541	209	100,0	100,0	100,0	135	596	23	100,0	100,0	100,0
	Exclusive Parental UP	149	658	43	100,0	100,0	100,0	131	850	3	100,0	100,0	100,0
	Exclusive Derived DW	253	235	343	100,0	100,0	100,0	255	8	323	100,0	100,0	100,0
	Exclusive Derived UP	500	42	645	100,0	100,0	100,0	494	1	638	100,0	100,0	100,0
	Common DW	42	76	86	100,0	100,0	100,0	33	8	12	100,0	100,0	100,0
	Common UP	24	37	20	100,0	100,0	100,0	15	9	1	100,0	100,0	100,0
	Common Parental DW Derived UP	453	28	300	100,0	100,0	100,0	378	4	18	100,0	100,0	100,0
	Common Parental UP Derived DW	343	287	44	100,0	100,0	100,0	319	35	10	100,0	100,0	100,0
**Proliferative signaling**	Exclusive Parental	2	1	0	100,0	100,0	0,0	2	0	0	100,0	0,0	0,0
	Exclusive Derived	1	2	3	0,0	100,0	100,0	1	0	0	0,0	0,0	0,0
	Continuum	185	125	23	70,8	75,2	78,3	134	0	1	72,4	0,0	0,0
	Exclusive Parental DW	85	236	110	87,1	69,1	70,0	77	265	15	87,0	67,2	73,3
	Exclusive Parental UP	90	367	23	67,8	73,0	82,6	77	514	2	68,8	74,1	100,0
	Exclusive Derived DW	115	125	207	71,3	64,8	75,8	114	8	202	70,2	62,5	77,7
	Exclusive Derived UP	292	23	329	71,6	87,0	69,6	302	1	347	72,2	0,0	67,4
	Common DW	26	39	66	73,1	53,8	68,2	22	6	12	72,7	50,0	58,3
	Common UP	17	25	13	64,7	88,0	76,9	14	7	1	64,3	100,0	100,0
	Common Parental DW Derived UP	267	17	161	78,3	76,5	65,2	227	2	12	78,0	100,0	91,7
	Common Parental UP Derived DW	149	181	29	63,1	74,0	89,7	139	26	7	63,3	73,1	85,7
**Evading growth suppressors**	Exclusive Parental	0	2	0	0,0	100,0	0,0	0	0	0	0,0	0,0	0,0
	Exclusive Derived	1	0	3	100,0	0,0	100,0	1	0	0	100,0	0,0	0,0
	Continuum	143	98	19	74,8	76,5	73,7	101	0	1	76,2	0,0	0,0
	Exclusive Parental DW	63	160	89	92,1	76,9	73,0	54	179	10	90,7	76,0	80,0
	Exclusive Parental UP	70	266	16	64,3	71,8	87,5	60	378	1	65,0	73,3	100,0
	Exclusive Derived DW	85	97	154	75,3	67,0	74,7	86	6	143	77,9	66,7	77,6
	Exclusive Derived UP	222	15	230	71,2	86,7	72,2	231	1	240	71,9	0,0	69,2
	Common DW	15	23	43	73,3	56,5	72,1	13	4	7	69,2	50,0	71,4
	Common UP	12	18	8	58,3	94,4	87,5	10	4	0	60,0	100,0	0,0
	Common Parental DW Derived UP	182	10	114	78,0	80,0	64,9	158	1	10	78,5	100,0	90,0
	Common Parental UP Derived DW	90	136	19	73,3	75,0	89,5	86	17	5	74,4	82,4	80,0
**Resisting cell death**	Exclusive Parental	0	3	0	0,0	100,0	0,0	0	0	0	0,0	0,0	0,0
	Exclusive Derived	1	2	2	100,0	50,0	100,0	1	0	0	100,0	0,0	0,0
	Continuum	232	137	28	67,2	70,1	75,0	164	0	0	68,3	0,0	0,0
	Exclusive Parental DW	65	236	115	78,5	64,4	76,5	67	264	12	71,6	63,3	58,3
	Exclusive Parental UP	103	304	16	57,3	72,4	93,8	94	436	1	55,3	75,0	100,0
	Exclusive Derived DW	111	157	177	63,1	66,2	74,6	109	7	163	64,2	42,9	75,5
	Exclusive Derived UP	240	13	331	76,7	100,0	64,7	260	1	357	76,2	100,0	60,8
	Common DW	20	33	40	70,0	57,6	85,0	16	2	7	62,5	100,0	71,4
	Common UP	17	23	8	70,6	73,9	75,0	11	3	0	72,7	100,0	0,0
	Common Parental DW Derived UP	214	9	188	72,4	66,7	62,8	181	1	4	73,5	100,0	75,0
	Common Parental UP Derived DW	151	164	21	62,3	76,8	71,4	143	14	4	63,6	71,4	100,0
**Replicative immortality**	Exclusive Parental	0	0	0	0,0	0,0	0,0	0	0	0	0,0	0,0	0,0
	Exclusive Derived	0	0	1	0,0	0,0	100,0	0	0	0	0,0	0,0	0,0
	Continuum	136	85	17	58,1	63,5	70,6	95	0	1	58,9	0,0	0,0
	Exclusive Parental DW	43	190	63	81,4	57,9	61,9	38	212	6	73,7	57,5	83,3
	Exclusive Parental UP	68	199	11	47,1	60,8	72,7	59	278	1	44,1	60,8	100,0
	Exclusive Derived DW	90	83	101	55,6	51,8	69,3	94	3	97	57,4	33,3	72,2
	Exclusive Derived UP	170	10	233	58,2	70,0	53,6	246	1	235	56,9	0,0	51,1
	Common DW	9	19	27	55,6	52,6	77,8	10	1	4	50,0	100,0	50,0
	Common UP	11	11	9	63,6	81,8	77,8	9	2	0	44,4	100,0	0,0
	Common Parental DW Derived UP	123	10	104	65,9	60,0	46,2	114	0	6	67,5	0,0	100,0
	Common Parental UP Derived DW	117	98	11	58,1	59,2	63,6	110	12	2	59,1	83,3	50,0
**Energy metabolism**	Exclusive Parental	4	0	2	25,0	0,0	0,0	2	0	1	50,0	0,0	0,0
	Exclusive Derived	3	3	2	33,3	66,7	50,0	3	0	0	33,3	0,0	0,0
	Continuum	338	200	56	51,2	58,0	53,6	232	0	5	51,3	0,0	20,0
	Exclusive Parental DW	120	626	147	59,2	48,7	54,4	115	674	22	60,0	47,9	59,1
	Exclusive Parental UP	137	558	29	48,2	61,5	69,0	121	701	1	47,1	60,1	100,0
	Exclusive Derived DW	291	183	294	47,8	49,7	58,2	299	13	275	49,2	38,5	56,7
	Exclusive Derived UP	374	31	638	64,4	58,1	53,0	359	1	612	64,6	0,0	53,6
	Common DW	30	61	75	56,7	36,1	49,3	27	4	10	51,9	0,0	20,0
	Common UP	24	29	13	58,3	62,1	69,2	17	4	1	47,1	100,0	0,0
	Common Parental DW Derived UP	379	22	221	59,4	50,0	56,1	325	1	11	55,1	100,0	72,7
	Common Parental UP Derived DW	386	203	39	46,1	64,0	46,2	358	26	4	45,3	61,5	100,0
**Other cancer types**	Exclusive Parental	2	0	0	50,0	0,0	50,0	1	0	0	0,0	0,0	0,0
	Exclusive Derived	2	0	2	0,0	0,0	0,0	0	0	0	0,0	0,0	0,0
	Continuum	85	65	20	61,2	69,2	68,0	68	0	0	64,7	0,0	0,0
	Exclusive Parental DW	32	100	43	68,8	61,0	73,7	27	115	6	70,4	59,1	100,0
	Exclusive Parental UP	30	180	10	60,0	71,7	66,7	26	253	2	57,7	70,8	100,0
	Exclusive Derived DW	47	52	88	63,8	57,7	66,7	47	1	93	63,8	100,0	73,1
	Exclusive Derived UP	148	9	137	74,3	55,6	75,8	152	0	158	77,0	0,0	58,2
	Common DW	15	15	24	73,3	40,0	64,7	12	1	2	50,0	0,0	50,0
	Common UP	6	9	2	33,3	77,8	42,9	7	2	0	57,1	100,0	0,0
	Common Parental DW Derived UP	127	5	71	60,6	80,0	62,2	108	0	6	19,4	0,0	83,3
	Common Parental UP Derived DW	61	94	8	50,8	69,1	54,3	58	12	0	56,9	83,3	0,0
**Chronic diseases**	Exclusive Parental	4	1	0	75,0	100,0	0,0	3	0	0	100,0	0,0	0,0
	Exclusive Derived	1	1	2	100,0	100,0	50,0	1	0	1	100,0	0,0	0,0
	Continuum	172	128	27	65,1	68,8	59,3	130	0	0	65,4	0,0	0,0
	Exclusive Parental DW	98	245	80	67,3	50,6	75,0	88	268	6	73,9	50,4	83,3
	Exclusive Parental UP	87	353	23	62,1	70,3	60,9	84	456	0	58,3	71,9	0,0
	Exclusive Derived DW	115	100	176	53,0	74,0	70,5	114	3	172	50,9	100,0	72,1
	Exclusive Derived UP	249	29	343	75,1	58,6	58,3	258	1	331	76,0	0,0	63,1
	Common DW	24	32	47	62,5	53,1	72,3	20	5	7	60,0	20,0	28,6
	Common UP	15	20	9	66,7	80,0	66,7	10	4	1	70,0	75,0	0,0
	Common Parental DW Derived UP	239	13	133	69,9	61,5	71,4	203	2	6	67,5	100,0	100,0
	Common Parental UP Derived DW	151	133	29	47,0	75,9	72,4	142	11	6	48,6	72,7	83,3
**Angiogenesis**	Exclusive Parental	2	2	0	100,0	100,0	0,0	2	0	0	100,0	0,0	0,0
	Exclusive Derived	1	3	0	100,0	100,0	0,0	1	0	0	100,0	0,0	0,0
	Continuum	118	81	19	82,2	87,7	89,5	84	0	1	82,1	0,0	0,0
	Exclusive Parental DW	71	178	61	88,7	73,0	83,6	67	189	8	92,5	75,1	100,0
	Exclusive Parental UP	43	297	20	86,0	85,2	100,0	40	387	3	82,5	85,3	100,0
	Exclusive Derived DW	93	57	169	77,4	91,2	85,2	88	5	162	73,9	100,0	87,7
	Exclusive Derived UP	210	18	233	83,8	94,4	78,1	213	2	234	85,4	50,0	82,1
	Common DW	24	21	45	91,7	90,5	86,7	20	4	7	90,0	75,0	85,7
	Common UP	12	24	13	75,0	91,7	92,3	8	9	3	75,0	88,9	33,3
	Common Parental DW Derived UP	219	12	94	87,2	100,0	90,4	188	2	7	86,2	100,0	100,0
	Common Parental UP Derived DW	95	122	21	71,6	86,1	95,2	84	13	7	72,6	92,3	100,0
**Evading immune destruction**	Exclusive Parental	2	3	0	50,0	100,0	0,0	1	0	0	0,0	0,0	0,0
	Exclusive Derived	2	2	2	100,0	100,0	100,0	2	0	0	100,0	0,0	0,0
	Continuum	211	137	32	80,6	85,4	96,9	152	0	0	82,2	0,0	0,0
	Exclusive Parental DW	92	301	122	87,0	86,0	75,4	86	330	7	87,2	83,0	85,7
	Exclusive Parental UP	83	404	21	79,5	89,6	81,0	75	533	1	81,3	89,3	100,0
	Exclusive Derived DW	157	114	219	80,9	82,5	89,0	150	4	215	82,0	75,0	87,4
	Exclusive Derived UP	287	27	368	90,9	81,5	86,7	284	2	336	89,8	50,0	87,8
	Common DW	20	43	55	90,0	67,4	94,5	15	1	6	86,7	100,0	100,0
	Common UP	15	26	9	86,7	88,5	100,0	9	6	2	88,9	100,0	50,0
	Common Parental DW Derived UP	283	13	135	88,7	76,9	85,9	243	2	9	88,5	100,0	88,9
	Common Parental UP Derived DW	185	165	35	84,3	89,1	82,9	173	17	7	83,8	88,2	100,0

DW: down regulated; UP: up regulated, for Clusters of Gene Expression.

Summary of cell line comparisons:

#### ZsG *vs*. LN1

The exclusive LN1 up regulated CoGE within the invasion and metastasis hallmark displayed the highest number of DEGs (500 for *p-*values and 494 for *q*-values). The lowest number of DEGs was related to other cancer types and replicative immortality. The hallmarks with highest contributions to invasion and metastasis were angiogenesis, evasion of immune destruction and evasion of growth suppressors, whereas the lowest contributions were energy metabolism and other cancer types. Results suggested that ZsG cells displayed the highest proliferative capacity and resembled “neurodegenerative diseases” more closely than LN1 cells. In contrast, LN1 cells were matched to other cancer types besides having a different cytoskeleton regulation and interactions with the extracellular matrix (ECM) than ZsG cells (Supplementary Tables 7A and 7B and Table 2).

#### LN1 *vs*. LN2

The CoGE displaying the highest number of DEGs was exclusive in LN1 up regulated concerning the invasion and metastasis hallmark for both *p-* and *q-*values, represented by 658 and 850 genes, respectively. The lowest numbers of DEGs were related to other cancer types and replicative immortality, displaying some CoGE without DEGs. The highest hallmarks contributions were evading immune destruction, angiogenesis, proliferative signaling and resisting cell death, while the lowest corresponded to energy metabolism and other cancer types. We concluded based on the KEGG pathways of each CoGE, that both LN1 and LN2 cell lines had features associated with the invasive process although resorting to different mechanisms, in which LN1 cells used inflammatory and proliferative processes to become invasive, whereas LN2 cells relied on strategies to become refractory to the immune system (Supplementary Tables 7A and 7B and Table 2).

#### LN2 *vs*. LN3

The CoGE exclusive LN3 up regulated displayed the highest number of DEGs for *p*-values (645) and *q-*values (638). The lowest numbers of DEGs were related to other cancer types and replicative immortality, with some CoGE without DEGs. Following invasion and metastasis, the hallmarks most represented were evading immune destruction, angiogenesis, evading growth suppressors and proliferative signaling in that order. Those with the least contributions were energy metabolism and other cancer types. Based on the KEGG pathways for each CoGE, we suggest that LN2 cells, rather than LN3 cells, showed a remarkable similarity to other cancer types, while the LN3 cell line was closer to high invasive capacity through angiogenesis stimulation and ability to avoid the immune system (Supplementary Tables 7A and 7B and Table 2).

**Figure 3:**
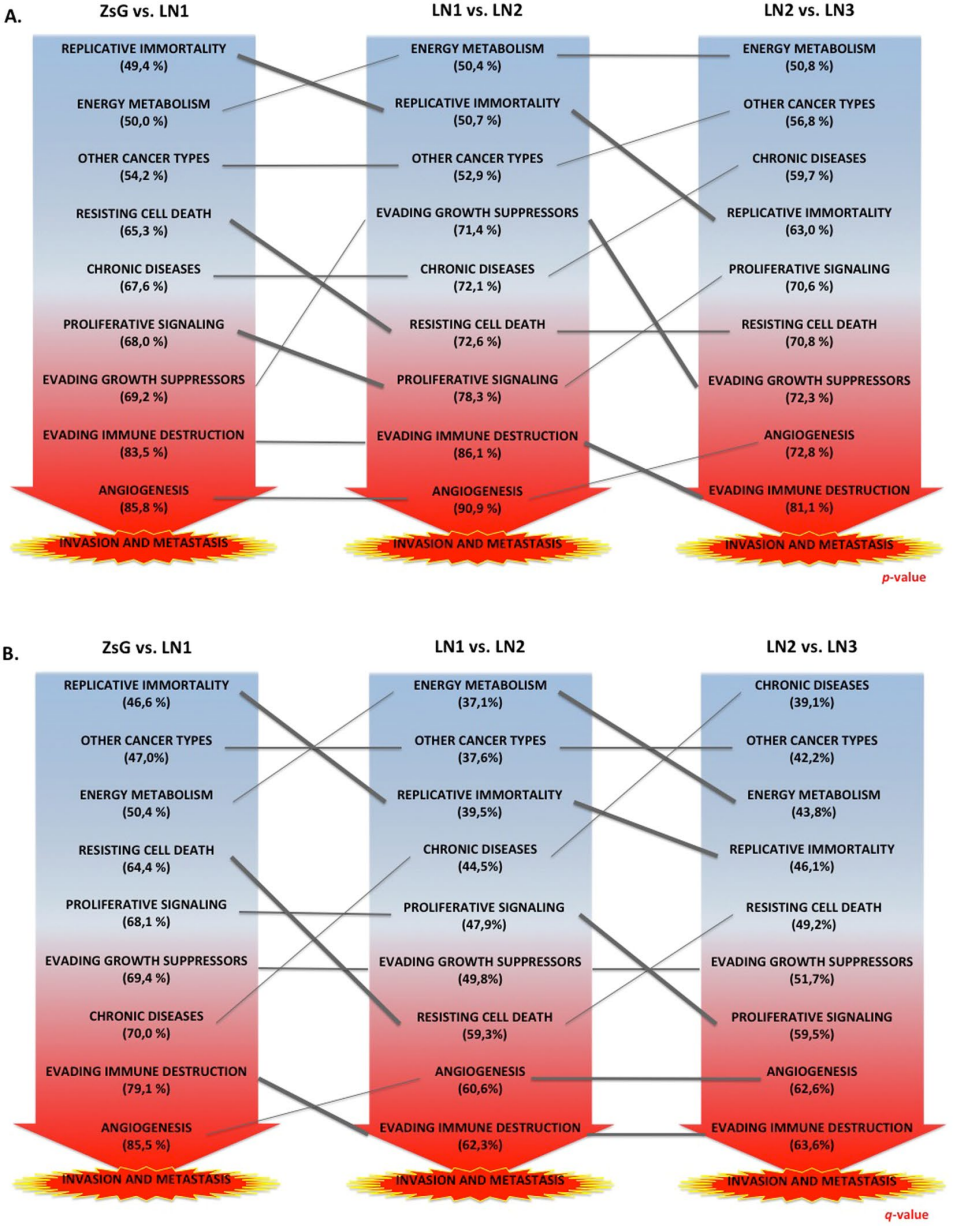
Individual contributions and percentages, (in parenthesis), of each hallmark to the invasive process. The total percentages are the mean of the 11-percentages of the clusters of gene expression (CoGE) for each hallmark, for (**A**) *p-*values, and (**B**) *q-*values.

In order to identify the global contributions of each hallmark of cancer to invasion and metastasis, we obtained the mean of percentages of 11-CoGE and ranked them accordingly (Fig. 3). We found that the processes most closely related to invasion and metastasis were angiogenesis, evading immune destruction, evading growth suppressors and proliferative signaling, with genetic contributions from more than 68% for *p-*values and almost 48% for *q-*values. Energy metabolism and other cancer types were less relevant, with contributions of almost 50% and 37% of their DEGs (*p-* and *q*-values comparisons, respectively) (Table 2).

### MYH14, ANGPTL4, ENPP1 and PPARD are possible OTSCC biomarkers and potential targets for interference studies

Next we looked for specific genes in our model trying to pinpoint novel OTSCC or metastasis biomarkers. Accordingly, we checked 11-CoGE of each cell line comparison, finding 26 common DEGs displaying the same type of regulation (Table 3) along with the OTSCC model of increasing metastatic potential. They were sorted as 15-downregulated, 10-continuum and 1 up regulated. These genes were classified using Panther^®^ software to analyze whether they were eligible as biomarkers for OTSCC and/or for metastasis.

**Table 3:** Common DEGs displaying altered expression and their regulation into our invasive progression model of tongue cancer and their FPKM expression mean for each cell line.

GENE ID	ZsG	LN1	LN2	LN3
**Common Down Reguated**				
MYH14	0,4972	0,2182	0,0534	0,0069
**RSAD2**	**28,3575**	**20,9164**	**8,3588**	**1,1869**
SLC28A3	0,7201	0,3187	0,1672	0,0511
LIPH	1,4704	0,896	0,5748	0,0333
GJA5	3,9214	0,7377	0,2756	0,1825
FGD3	0,8897	0,4476	0,1145	0,0305
**VGLL1**	**5,1019**	**2,0341**	**0,6328**	**0,0501**
RAB17	1,4872	1,0823	0,6931	0,1211
PLXDC2	0,5215	0,1356	0,0163	0,001
NMU	46,4355	34,3583	25,9269	3,6182
**SCEL**	**2,7007**	**1,4439**	**0,4708**	**0,0844**
SCNN1A	60,9356	35,3696	20,6939	4,5639
UNC5B	4,2487	3,189	2,3056	0,2049
**ANGPTL4**	**25,9462**	**11,4337**	**7,7907**	**5,4245**
CXADR	2,0463	1,6011	0,9881	0,5827
**Continuum**				
DAPK3	27,8868	23,4451	19,0566	22,2199
STX6	15,4365	13,654	11,4419	9,341
CHMP6	20,0775	18,2558	15,6105	18,5514
SRPRB	33,1723	30,4336	25,6505	29,8296
DIABLO	30,5994	32,3878	28,2071	33,1907
PIGG	13,7416	15,1826	12,9338	14,9107
TMED2	195,729	217,1806	178,7479	213,2503
TRPC4AP	30,9148	36,4274	30,1783	35,0563
**PPARD**	**11,7148**	**13,8269**	**11,7824**	**10,1059**
SLC8B1	14,8684	15,557	12,9781	10,6452
**Common Up Regulated**				
**ENPP1**	**1,1246**	**1,4489**	**2,0885**	**5,2621**

The remarked genes were selected to validate by real time PCR.

First, we concentrated on the 15-downregulated genes, and found that only angiopoitein related protein 4 (ANGPTL4) was associated to biological adhesion acting as a signaling molecule. Amiloride-sensitive sodium channel subunit alpha (SCNN1A) and Ras-related protein Rab-17 (RAB17) participate in biological regulation. Myosin 14 (MYH14), which acts as G-protein modulator by way of the actin binding motor protein and as a cell junction protein, as well as RAB17, are related to cellular component organization and biogenesis. Regarding cellular processes, six genes, solute carrier family 28 member 3 (SLC28A3), gap-junction alpha-5 protein (GJA5), netrin receptor UNC5B (UNC5B), ANGPTL4, MYH14, and RAB17 were detected. Five genes were associated to developmental processes, namely ANGPTL4, MYH14, UNC5B, transcription cofactor vestigial-like protein 1 (VGLL1) and FYVE, RhoGEF and PH domain-containing protein 3 (FGD3), which acts as guanyl-nucleotide exchange factor. Four genes were related to cellular localization, SCNN1A, SLC28A3, MYH14 and RAB17. Concerning metabolic processes, we detected lipase member H (LIPH), which acts as esterase, phospholipase and storage protein. Two genes were related to multicellular organismal process, SCNN1A and MYH14. LIPH was associated to cell proliferation and motility. Within the group of 15-downregulated genes, ANGPTL4 is a classical biomarker for metastasis. Five genes were not found in the Panther database. Therefore, we used Gene Ontology to find out their biological functions. Two of them had receptor functions, plexin domain containing 2 (PLXDC2) and neuromedin U (NMU), that acts as neuromedin U receptor binding. Other two genes with binding properties, radical S-adenosyl methionine domain containing 2 (RSAD2) acting as a self-association protein and as iron-sulfur cluster binding, and coxsackie virus and adenovirus receptor (CXADR) identical protein binding and integrin binding. Finally, sciellin (SCEL) takes part in the assembly or regulation of proteins in the cornified envelope.

The single up regulated gene, ENPP1, was classified as part of the metabolic process, being a nucleotide phosphatase and pyrophosphatase enzyme.

Inspection of the ten continuum genes revealed one gene related to cellular component organization or biogenesis, syntaxin-6 (STX6), which acts as a SNARE protein. Four genes were related to cellular processes, signal recognition particle receptor subunit beta (SRPRB), peroxisome proliferator-activated receptor delta (PPARD), GPI ethanolamine phosphate transferase 2 (PIGG) and STX6. Four genes were classified within the localization group: charged multivesicular body protein 6 (CHMP6) that acts as transfer/carrier protein; transmembrane emp24 domain containing protein 2 (TMED2), which acts as transfer/carrier protein and as vesicle coat protein; SRPRB and STX6. Also, 2 genes related to metabolic process, PPARD and PIGG were found. Only one gene was related to multicellular organismal process, PPARD, a member of the proliferator-activated receptor family PPAR involved in the development of several chronic diseases. Four genes were not found in the Panther database, so we used Gene Ontology to search for their biological functions. Two were related to cell death, death associated protein kinase 3 (DAPK3), which displays protein homodimerization activity and transferase activity of phosphorus-containing groups; and second mitochondria-derived activator of caspase (SMAC/DIABLO), which activates caspases by binding to inhibitor of apoptosis proteins. Two genes were related to transport functions, sodium/potassium/calcium exchanger 6 mitochondrial (SLC8B1) acting as a cation transporter, and transient receptor potential cation channel subfamily C member 4 associated protein (TRPC4AP), related to phosphatase binding.

**Figure 4:**
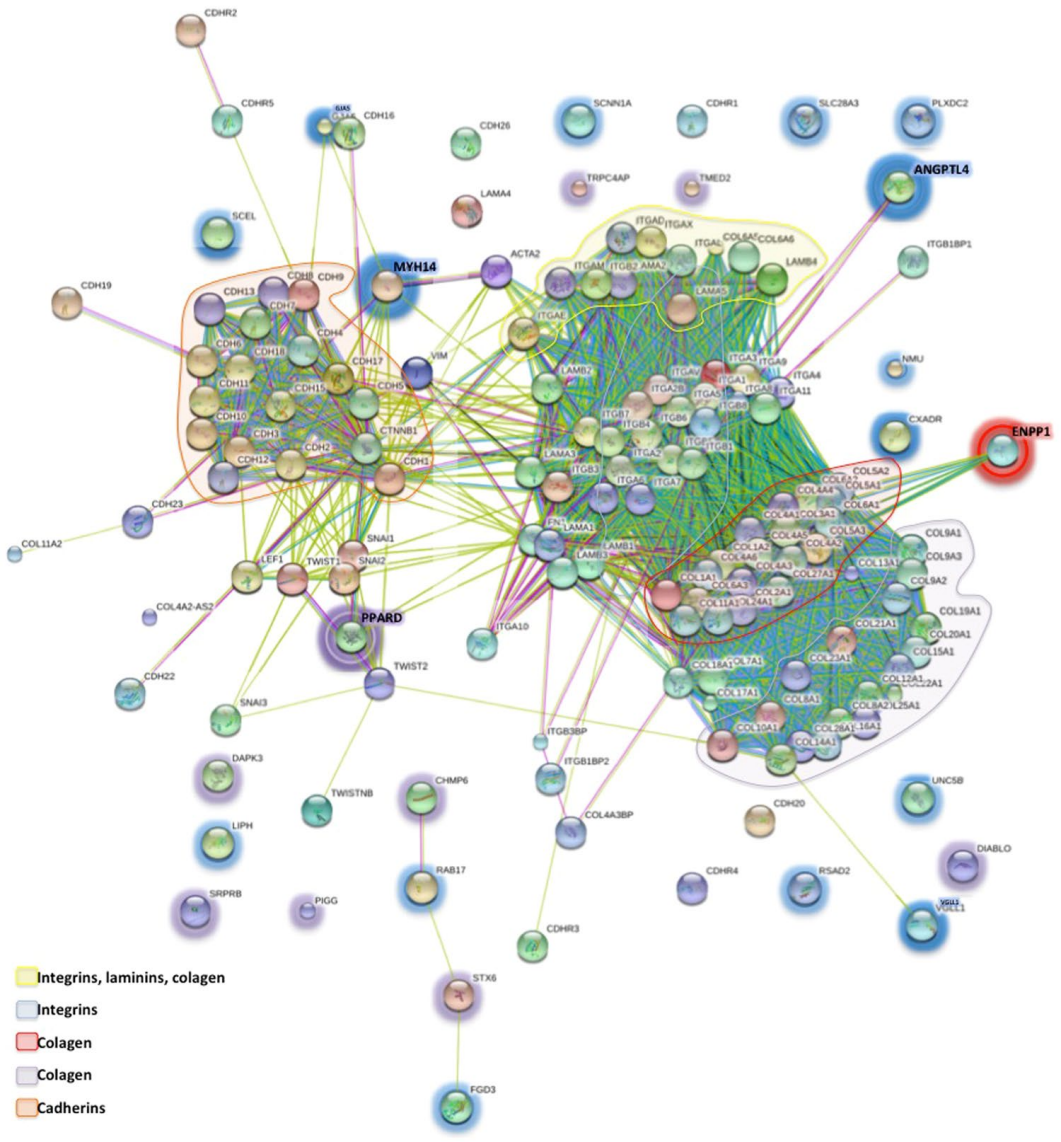
Interactome showing the contributions of the 26-common DEGs displaying altered expression of the metastatic model of OTSSC to the consensual biomarkers associated to epithelial-mesenchymal transition. Highlighted circles represent the common DEGs displaying altered-expression detected in our transcriptome analysis. Highlighted circles in blue are down regulated genes, purple, continuum genes and red, up regulated genes.

To better understand the relationship between the above 26 highlighted genes, we investigated whether they interacted with each other using STRING. Four genes RAB17, FGD3 (down regulated), STX6 and CHMP6 (continuum) (Fig. 4) were found to be linked.

Based on the most common biomarkers of squamous cell carcinoma reviewed by Scanlon *et al*.^13^, we selected all the expressed genes related to epithelial-mesenchymal transition (EMT) in our OTSCC model (Supplementary Table S7). In order to understand the relationship between those genes, we used STRING, which displayed 5 sub-clusters, grouping as (i) cadherins, (ii) laminins, collagen and integrins, (iii) integrins and laminins, and two clusters of collagen (iv, v) (Fig. 4). Interestingly, when we analyzed those genes grouping with our list of 26 highlighted genes, we found that the up regulated gene ENPP1 clustered with a collagen sub-cluster; ANGPTL4, a down regulated gene grouped with the integrins of a sub-cluster of laminins and integrins. MYH14, another down regulated gene, grouped with the sub-cluster of cadherins. Finally, PPARD, a gene of the continuum group, clustered with fibronectin and the sub-cluster of laminins, also exhibited higher affinity for the transcription factors SNAIL1 and SNAIL2, TWIST and LEF1, the most important transcription factors of the EMT of HNSCC^13^. Therefore, those 4 genes stand out as potential targets for oral cancer therapy.

**Figure 5:**
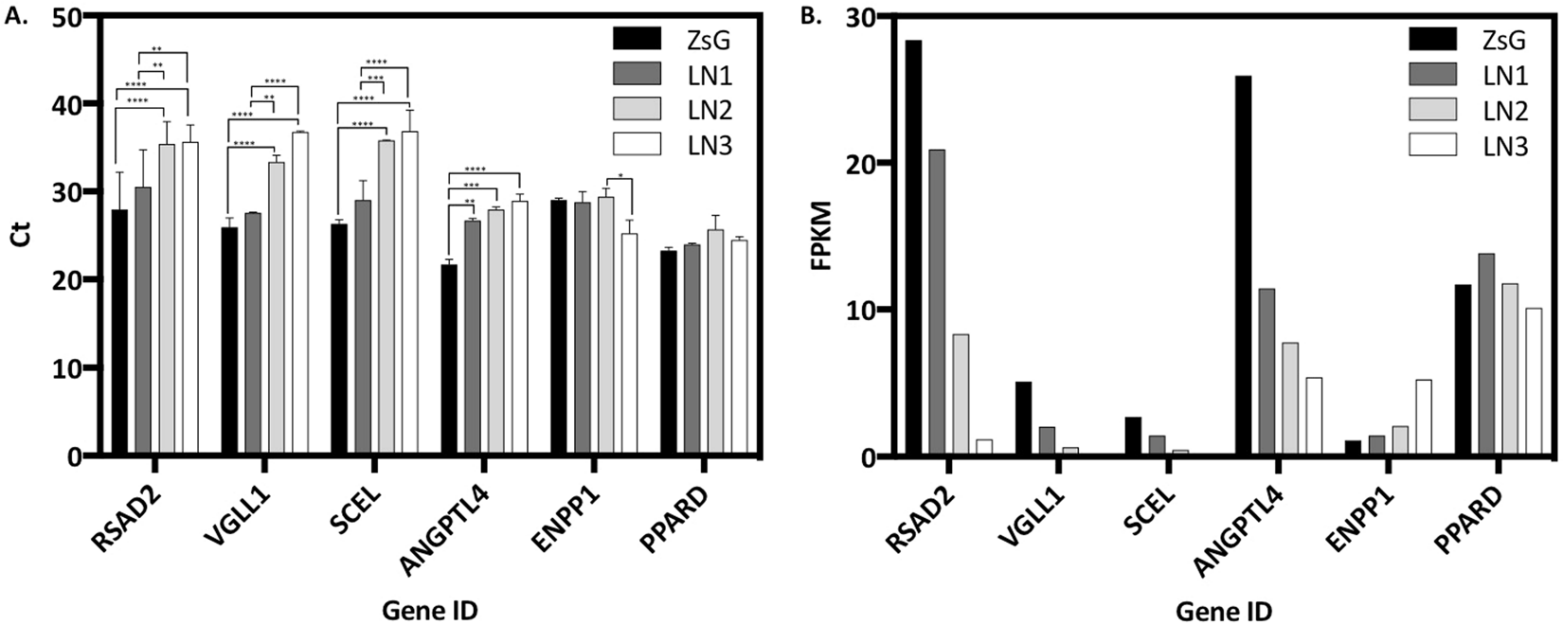
Expression profiles of the selected common DEGs displaying altered-expression for each cell line, of 3 independent experiments. (**A**) RT-PCR data and (**B**) FPKM data. Turkey’s multiple comparisons test, ****p < 0.0001; ***p < 0.001; **p < 0.01; *p < 0.05.

To validate some of the common DEGs displaying altered expression, we selected 4-down regulated, 1-continuum and 1-up regulated genes (Table 3, highlighted genes). We carried out RT-PCR and plotted the results relative to Ct (threshold cycle), as well as to the transcriptomic data (FPKM) (Fig. 5). The results showed that down regulated genes enhance their Ct values along with the metastatic progression. This means that less aggressive cells display higher expression, whereas the most aggressive stages exhibited a lower degree of expression; in contrast, up regulated gene ENPP1 displayed a continuum pattern, as well as PPARD, a continuum gene.

### Clinical data is consistent with our 4 potential therapeutic targets expression

**Figure 6:**
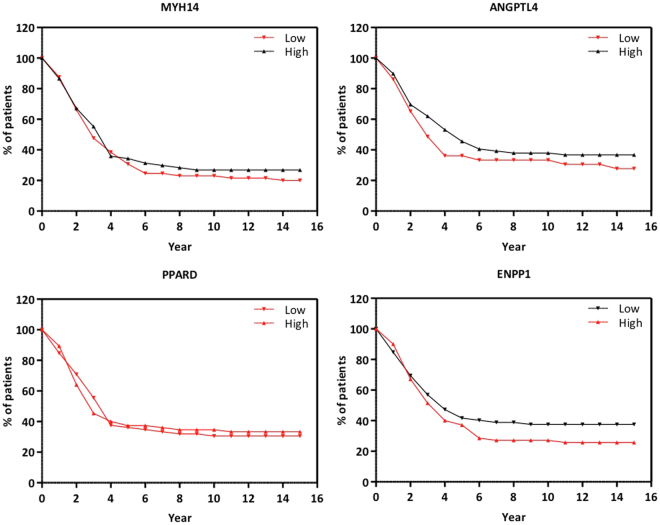
Clinical data of head and neck cancer from TCGA (n = 248), relative to proposed molecular targets. Low expression of (**A**) MYH14 and (**B**) ANGPTL4 (red line) and high expression (black line); (**C**) low and high expression (red lines) of PPARD; and high (red line) and low expression (black line) of ENPP1, in alive patients. Dotted gray lines represent gene expression of dead patients.

In order to highlight the expression of these 4 potential therapeutic genes in patients of head and neck cancer, data from The Cancer Genome Atlas (TCGA, https://cancergenome.nih.gov/) of 248 clinical tumors were used to support our observations. In this regard, we analyzed the 4 genes proposed as possible molecular targets (ANGPTL4, MYH14, PPARD and ENPP1, Fig. 6, red lines) in terms of levels of gene expression: low, continuum and high. Considering that metastasis is the major factor in cancer lethality, we found a remarkable correlation between up and down regulation of selected genes and the survival rate of cancer patients. Low expression of genes ANGPTL4 and MYH14 correlates with high lethality (Fig. 6A and B, in red). Nevertheless, the up regulation of gene ENPP1 correlates with high lethality as shown in Fig. 6C, in red. In addition, gene PPARD whose expression did not change (continuum) appears to have no correlation with the survival rate plots (Fig. 6D, in red).

## Discussion

Here, we sequenced and analyzed the transcriptomic data of 5 cell lines of OTSCC, characterized by their progressively increasing invasive capacity. The sequencing was made for 3 independent experiments for each cell line. Similar studies^14,15^ have reported 21000 expressed genes. In our screening we found 28000 genes. The significant difference may be ascribed to the approach employed in the present work, namely pooling together seven data sets obtained from the biological and technical replicates (Supplementary Table 1). One way to validate the consistency of our datasets was to rank the genes according to the level of expression (FPKM) and to compare whether the top 10 most expressed genes were comparable taking into account both for the independent experiments and the experimental replicates (7 datasets). We found a high correlation that showed 6 highly expressed genes in LN1 and LN2 cell lines, 7 in LN3 cells and 8 in SCC9 and ZSG cell lines, respectively (data not shown). For all cell lines and their replicates microRNA6723 was the most expressed gene in each one of the 35 datasets. Also, we found that among the top 10 expressed genes the calcium binding proteins S100A6 and S100A9 proteins were included. S100A9 protein were associated to chronic inflammation in hypoxia response^16^, a possible mechanism that OTSCC induce to develop more aggressive stages.

In order to detect the differentially expressed genes (DEGs), we compared the parental cells with its derived cell line applying the student’s *t*-test (*p-*values to the expression values). We found more than 5.000 DEGs. Then, we corrected those data by applying the FDR correction (*q*-values) to minimize the type I error. We found values between 284 and 9.874 (Supplementary Table 2). Usually transcriptomic analyses are based on *p*-values. However, many reports are based on *q*-values to reduce the number of genes to be analyzed functionally. The problems involved in this kind of analyses are the cut-offs based on fold change and statistical significance^9^. Thus, depending on the aim of the work, one has to establish a compromise between the significance of the functional attributes of the gene expression and the number of genes analyzed. On the other hand, many works in the literature have “cleaned” their data by eliminating many non-coding genes (NCG) that have not part of specific biochemical pathways^17^. In the same way, some parameters that take into account the most differentially expressed genes, assume that the complexity of the cells could be downscaled to a small number of genes. In this work, we used all DEGs for *p-* and *q-*values and compare them in order to show the relative importance of this correction, holding a comprehensive and more robust analysis of the complex cell biological systems.

By comparing DEGs between the parental and its derived cell line, we found 11-CoGE (Supplementary Tables 3–6). They display all the possible types of expression regulation for each gene and for each comparison of the transformed cell lines. Establishing CoGE is an interesting tool to find the common DEGs and their expression properties between the comparisons. With this approach, new OTSCC biomarkers were identified (Table 3). In addition, this strategy evidenced expression features that may have been acquired or lost between the compared cells. In other words, CoGE can reveal in greater detail those phenotypic traits of cell lines that display individual or common characteristics that fall within the general bracket of malignancy. Usually, the analysis tools displays patterns of gene expression, which include some of the 11-CoGE described here but not all of them^11,18–20^. In this report we have provided a comprehensive view of all gene regulatory possibilities when considering transcriptomics, as related to tumor progression.

DEGs for *p-* and *q-*values were enriched for the PCG with their related KEGG pathways (Table 1). The analysis allowed the observation that the number of genes considered did not necessarily produce a proportional number of pathways. This could be interpreted as meaning that genes may be endowed with multiple functions promoting in tumor cells highly plastic networks. An excess of PCGs resulting in fewer pathways could indicate a high functional redundancy. Accordingly, we found 284 KEGG pathways related to LN1 *vs*. LN2, exclusive DEGs up regulated in LN1 cells, for both *p-* and *q*-values. The groups comprising the commonly expressed genes (down or up regulated) had lower number of PCG and KEGG pathways. However, when analyzing the number of KEGG pathways related to the human genome, we used all-35238 annotated cDNAs following the same procedures, which yielded 292 KEGG pathways (Fig. 2, Supplementary Table 6C).

In order to find out the KEGG pathways related to the 8 hallmarks of cancer described by Hanahan and Weinberg in 2011^10^, we classified each KEGG pathway into each characteristic proposed for cancer by performing manual curation of the data in the literature. Additionally, we found also 71 KEGG pathways related to other cancer types and chronic diseases. For this reason we added these two categories to our analyses. We observed that energy metabolism was the hallmark that included the highest number of related KEGG pathways. Biological processes related to metabolism are frequently found as most altered pathways in large scale analyses^21–24^. After metabolism, the other hallmarks categories followed: invasion and metastasis, chronic diseases, proliferative signaling, resisting cell death and evading immune destruction, angiogenesis, evading growth suppressors, other cancer types and replicative immortality (Supplementary Table 6).

As our OTSCC model was developed by selecting a gradient of increasing metastatic potential, we used invasion and metastasis as a gold reference to weight the contributions of other hallmarks. Consequently, we compared the KEGG pathways falling into this category to KEGG pathways in the other hallmarks (Fig. 2 and Supplementary Table 7). Among these, 25 were shared with energy metabolism, 22 with immune destruction evasion, 18 with proliferative signaling; 13 with resistance to cell death and angiogenesis, 11 with growth suppression evasion; 3 with replicative immortality, 1 with other cancer types and 0 with chronic diseases.

Consistently, we used the related DEGs to each KEGG pathway, searching for the genetic contributions of each hallmark to invasion and metastasis. We found that the principal inputs of the hallmarks corresponded to those with highest number of pathways and DEGs, namely evading immune destruction (22 pathways, 1542 genes) and energy metabolism (25 pathways, 1536 genes) (Fig. 2). In the same way, the less represented KEGG pathways and DEGs were found to display the lowest contributions, relative to replicative immortality and other cancer types. Interestingly, chronic diseases, a hallmark with no shared pathways with invasion and metastasis, had the fifth higher contribution of DEGs to the invasive process (0 pathways and 1028 shared genes). These data show that considering exclusively the number of pathways could be misleading.

All comparisons of DEGs related to KEGG pathways were carried out for each CoGE. Analyzing the same parameters (pathways and genes) we found clues about the specific biological features of each cell line. It must be mentioned, however, that we have no information as to whether those PCG are actually translated. Notwithstanding, preliminary proteomic data, have confirmed that the transcriptomic data parallel the implied mechanisms as show by the pathways analyses (Cesari IM *et al*., in preparation). It can be consider the possibility that the genes could also be post-transcriptionally and post-translationally regulated. Taken together the most important findings were that ZsG elements were closest to neurodegenerative diseases and also displayed more features of proliferative cells than LN1 cells. Conversely, LN1 elements were more similar to other cancer types, besides having different cytoskeleton regulation and interactions with the extracellular matrix (ECM) (Supplementary Tables 7A and 7B and Table 2). This follows the observation that up regulated transcripts in cancer are down regulated in central nervous system (CNS) diseases and *vice versa*^20^. Indeed, another report revealed that up regulated genes in CNS disorders coded for low abundant proteins, and that the opposite occurred in cancer^25^. These observations may support the idea that the highest similarity with neurodegenerative illnesses occurs in the less aggressive cell lines. Furthermore, it is known that common features of metastasis involve MMPs genes, transcription factors, cyclooxygenases, chemokines, etc.^6,26^, especially those related to ECM interactions.

Based on those comparisons we can infer that LN1 and LN2 use different mechanisms to become metastatic; LN1 resorts to inflammation and proliferation and LN2 to immune system evasion. Indeed, inflammation was associated with amplification of the signaling loops that favor the metastatic cascade^27–29^. This is in agreement with previous reports showing that gene silencing was associated with tumor progression and metastasis. A point in case is MTA2 (metastasis tumor-associated protein 2) in glioma, in which it has been shown that proliferation and metastasis were inhibited^30^, while this gene was found to be upregulated in nasopharyngeal cancer^31^. In addition, the immune system can promote either activation or suppression of tumor growth, in a process known as “immunoediting”^32^. Some cancer cells present tumor antigens that lead to their elimination by the immune system. Alternatively during the process of immunoediting, they can lose those antigens due to either random genetic instability or in response to immune-induced inflammation^32,33^. Finally, LN2 cells were more similar the tumor cells classified as “other cancer types” than LN3 cells, whereas LN3 could induce angiogenesis and were capable to evade the immune system (Supplementary Table 7). Both mechanisms were already discussed for ZsG and LN2 cells.

The next step consisted in looking for the relative contribution of DEGs for each hallmark of cancer to invasion and metastasis. Table 2 showed that angiogenesis and evading immune destruction DEGs were the most representative. Regarding the number of genes, angiogenesis and evasion of immune destruction were the first and the fifth hallmarks with highest contributions, respectively. Conversely energy metabolism and other cancer types were those with lowest contributions of DEGs, being the second and the last hallmarks with higher number of genes, respectively. This means that evading immune destruction is a hallmark highly associated to invasion and metastasis in OTSCC due to the number of genes and its contribution to that process. In contrast, energy metabolism, the hallmark that displayed the highest number of KEGG pathways and the second highest number of gene contributions to invasion and metastasis, was less related to the invasive process (Fig. 3). These observations suggest that cancer therapies should target those genes involved in immune system evasion, angiogenesis and/or growth suppressors avoidance. It follows that although metabolism contains a high number of gene contributions, they may not be the most susceptible targets for an efficient therapy.

After evaluating the global behavior of the gene expression and its contributions to invasion, we identified common DEGs displaying altered-expression that could become biomarkers of OTSCC or metastasis. To accomplish that, we compared the 11 CoGE, and found 26 genes: 15 were down regulated, 10 were continuum and one up regulated (Table 3).

Of the subgroup of down regulated genes, we found 3 genes described as biomarkers of HNSCC (RAB17^34^, NMU^35^ and ANGPTL4^36^), and one of EMT (CXADR^37^). Of these, CXADR was reported to be down regulated^38^, agreeing with our results; no expression data for RAB17 was found although in our results we found to be down regulated; ANGPTL4 was reported to be overexpressed^39^ in contrast to our result showing it was down regulated; NMU protein was proposed as biomarker, although in our data measuring RNA/cDNA levels, this gene was down regulated. Other 4 genes have expression data in HNSCC, namely VGGL1^40^, SCEL^41^, SCNN1A^42^ and UNC5B^43^. In previous works all of them were found to be down regulated, in agreement with our analysis. Finally, 7 common DEGs which had not been reported before in association with HNSCC expression were found to be down regulated. These were MYH14, RSAD2, SLC28A3, LIPH, GJA5, PLXDC2, and FGD3. Interestingly, mutations for MYH14 have been described in HNSCC^44^ which were correlated to a negative regulatory activity of metastasis^45^. Incidentally, all 7 genes have been associated to cancer or metastasis and had been noted for their high level of expression^46–53^, even though PLXDC2 was down regulated in vulvar squamous cell carcinoma (VSCC). This was associated with unfavorable prognosis^54^.

In agreement with data obtained from renal carcinoma we found that DIABLO belonged to the continuum group^55^. Curiously this gene was found to be up regulated in cervical cancer^56^, in tumors of colorectal carcinoma patients^57^, and in gastrointestinal cancer^58^. In addition, TRPC4AP, another member of the continuum CoGE group did not display any type of regulation in mouse fibroblasts NIH-3T3 when induced by adenovirus early region 1 A protein (E1A) oncogene^11^, whereas it was found down regulated in a murine model of aggressive OSCC^59^. In the same way, PPARD, CHMP6 and TMED2 were found to be either down regulated^59–61^, or up regulated^61–63^ depending on the treatment and the cell line types. Only DAPK3 and STX6 had been reported to be expressed in HNSCC, being down^64^ and up regulated^65^. SRPRB was described as down regulated in peripheral blood cells of a melanoma patient when compared with healthy primary melanocyte cells^66^, as well as in breast cancer patients after 4 cycles of chemotherapy^67^. There are no data in cancer studies concerning SLC8B1 (encoding NCLX protein) and PIGG. However, SLC8B1 is known to play a key role in cellular and mitochondrial Ca^2+^ homeostasis and thereby, it is implicated in cell Ca^2+^ regulation, oxidative phosphorylation, hormonal secretion, synaptic transmission and apoptosis^68–70^. Moreover, PIGG is involved in ethanolamine phosphate transference and its mutations and deletions were reported to be associated with intellectual disorders, hypotonia and early-onset seizures^71^. Other members of PIGG’s family, such as classes U (PIGU), T (PIGT) and X (PIGX) are oncogenic, being overexpressed in bladder cancer^72^ and breast cancer cell lines^73,74^, suggesting a possible role in cancer development related to PIGG.

With regards to the up regulated genes the only one found was ENPP1 whose expression is stimulated by estrogen in stromal cells from normal human endometrium^75^. ENPP1 loss has been found in ovary cell lines occurring even without genomic deletion. The silencing of this gene can be attributed to hyper methylation of the connective tissue growth factor (CTGF/CCN2) promoter, that inhibits the expression of several genes^76^. Also, it was shown that ENPP1 is a potential facilitator of breast cancer bone metastasis, with high levels of both mRNA and protein synthesis^77^, occurring in a chromosomal region reported to be amplified in breast cancer^78^. The opposite was found in ovarian cell lines. Likewise it was shown that loss of microRNA-27b contributed to breast cancer stem cell generation by activating ENPP1. Clinical data suggest *ENPP1* expression in primary breast cancer tissues is associated with malignant potential and response to chemotherapy^79^. In HNSCC, it was reported that ENPP1 gene was activated by anti-inflammatory stimuli^80^.

Our observations on common DEGs displaying altered expression corroborate the findings regarding classical biomarkers of invasion, such as RAB17, NMU, ANGPTL4, CXADR and ENPP1, and also allowed the proposal for novel biomarkers of OTSCC metastasis, such as PIGG and SCL8B1 (Table 3). Furthermore, we found 24 of 26-common DEGs displaying altered expression related to different processes in many types of cancer, strengthening our analysis strategy. For instance, NMU gene that encodes a HNSCC biomarker was found to be down regulated at the RNA/cDNA level in our work, leading us to consider the occurrence of post-transcriptional and/or post-translational modifications. Quite probably, a certain number of genes that we have found to be differentially expressed may not synthesize proteins. Similarly the proteome profile may not be deduced from the transcriptomic data, due to many factors pertaining to the transcription process^81^. Our approach suggests a set of pathways, out of many possible ones, that OTSCC could possibly undertake to become more aggressive.

When we compared those 26 sequentially altered genes with traditional biomarkers for OSCC, we found that ANGPTL4, MYH14, ENPP1 and PPARD interact with important subsets of genes involved in EMT (Fig. 4). Collagen and integrins are important components of the ECM, and actively participate in the invasive process^82^. MYH14, ANGPTL4 and ENPP1 clustered with those genes, as observed in the interactome. The transcription factors SNAIL1, SNAIL2, TWIST and LEF-1 promote EMT in HNSSC^13^ and PPARD interacted with them. PPARD is activated by LEF-1^83^. This represents an interesting approach to define ANGPTL4, MYH14, ENPP1 and PPARD as novel HNSCC biomarkers. Moreover, we analyzed 248 clinical data of HNSCC from the TCGA and we found that expression levels of these genes in live patients correlate with our findings (Fig. 6).

Validation of the transcriptome was attempted by carrying out RT-PCR for 6 of the common DEGs displaying altered expression. Among them, 4 were down regulated in our model, and displayed a pattern that corroborated the transcriptomic data. Therefore, RSAD2, VGLL1, SCEL and ANGPTL4 could represent biomarkers of oral metastasis. On the other hand, ENPP1, an up regulated gene, did not display the same pattern as that of the RNA-Seq data. PPARD, a gene belonging the continuum CoGE, consistently did not change its expression amongst the metastatic progression (Table 3 and Fig. 5).

Finally, the reasoning used here for large-scale RNA-Seq analyses using all the DEGs with or without corrections (*p*- or *q*-values), in order to have a comprehensive and robust view of the complex cell system biology. No single analysis pipeline can be used in all cases^84^. The pipeline took into account PCG and KEGG pathways related to them. This allowed us to classify the DEGs related pathways into the hallmarks of cancer and to establish their contributions to any specific process or characteristic. In our case, we found that evading immune destruction and angiogenesis were the most related to invasion and metastasis. We propose that cancer treatments should be directed against those genes rather than metabolic or generic ones. Our approach can be used for other cellular and cancer related processes and diseases, being an interesting tool to highlight nodal genes or set of proteins on which to base new therapies. Lastly, genes such as ANGPTL4, MYH14, PPARD and ENPP1 might constitute interesting molecular targets for OTSCC treatment trials.

## Material and Methods

### Cell lines

Cell lines SCC9, and transformed ZsG, LN1, LN2 and LN3 were a kind gift from Agostini and collaborators. For details of model development, see reference^7^.

### Cell culture

Cells were cultured in Ham’s F12 medium (DMEM/F12; Invitrogen, USA) supplemented with 10% fetal bovine serum (FBS) and hydrocortisone 400 ng/ml (Sigma-Aldrich, USA). 1.1 × 10^6^ cells of SCC9, ZsG and LN1; 1.5 × 10^6^ cells of LN2 and 2 × 10^6^ cells of LN3 were transferred to 60.1 cm^2^ Petri dishes, for 48 hours, using an incubator series 8000 water-jacketed CO_2_ (Thermo Scientific), in humidity atmosphere of 5% of CO_2_. Three independent biological replicates of each cell line were used for the transcriptomic analysis. The cell lines were genotyped and tested free for *Mycoplasma sp*. infection.

### RNA extraction

Total RNA of ~6 × 10^6^ cells in 3 independent biological experiments for all 5 cell lines was extracted using RNeasy kit (Quiagen^®^), according to the manufacturer instructions. Quality and purity of the samples were quantified using Nanodrop ND1000 (Thermofisher Scientific).

### Library preparation and sequencing

Libraries were prepared using 4 μg of total RNA from each sample, strictly following the instructions of the TruSeq RNA Sample kit v2 (Illumina®). Seven technical replicates were obtained for all cell lines, from three biological independent experiments. Each library was uniquely identified using specific barcodes. The quality of library preparations was assessed using DNA 1000 kit for Bioanalyzer (Agilent®). Libraries were subsequently quantified by qPCR using Library quantification kit for Illumina (Kapa Biosystems^®^). 10 pM of sample libraries were distributed in 5 lanes of a flow cell, using TruSeq PE Cluster kit v3 - cBot – HS (Illumina®). A 100 × 100 Paired End run was carried out in an Illumina HiSeq2500® platform, using TruSeq™ SBS Kit v3 - HS - 200 cycles. Samples were multiplexed using Nextera kit, on which sequence adaptors were added into samples to differentiate and demultiplexed after the sequencing process.

### Data analysis

CASAVA^®^ tool was used to make the base calling, obtaining the FASTQ sequences for experimental and biological replicates. Using the FASTQ files, gMAP^®^ was employed to align our reads against the human genome v.38 (Ensembl), producing a GFF file and the Cufflinks tools align the coordinates of each read in GFF format to produce the frequency of each gene, expressed in Fragments Per Kilobase of exon per Million reads sequenced (FPKM). A FPKM ranking was obtained and these data were compared using Excel (Microsoft Corporation^®^). The first step consisted of the comparison between parental *vs*. its derived cell line, gene by gene, using Student’s *t*-test, to obtain the differentially expressed genes (DEGs). False Discovery Rate (FDR) correction was made, generating a *q*-value, reducing type I error. A ratio between the derived and parental cell lines was obtained, to determine the type of regulation for each gene, and to establish the clusters of gene expression (CoGE). STRING^®^ free software (http://string-db.org)^12^ was used to classify between protein coding genes (PCG) and non-coding genes, and to obtain the KEGG pathways related to each PCG. Panther^®^ (http://pantherdb.org) was used to determine the biological process of specific genes, based in gene ontology.

### RNA extraction and cDNA synthesis for validation experiments

Total RNA was isolated from oral cancer cells using TRIzol reagent (Invitrogen) according to the manufacturer’s instructions. Total RNA was quantified spectrophotometrically using Nanodrop ND1000 (Thermofisher Scientific) and 1 μg was treated with 1 unit of RNase-free DNase for 30 min at 37 °C. Reactions were stopped by adding 1 μl of 20 mM EDTA and heating for 10 min at 65 °C. Synthesis of cDNA was performed using the DNase-treated RNA according to a High Capacity cDNA Reverse Transcription Kit (Applied Biosystems).

### Real Time-PCR

Gene expression analysis was performed using a 7500 Real-Time PCR (Applied Biosystems) and power SYBR-Green PCR master mix (Applied Biosystems). The sequences of the primers used were: RSAD2 *forward* GCGTTGCGGGGAAACGAA *reverse* AGCGCCGGCCGTTTATC; VGLL1 *forward* GGACATCAGCAGCGTAGTGG *reverse* CTCTGACTCGAGGGGGTCAA; SCEL *forward* TTGCAACCTGGCGGTTCATT *reverse* ACACCTGGTTCCCTCTTCTTCT; ANGPTL4 *forward* CTCTCTGGAGGCTGGTGGTT *reverse* TGTGGGATGGAGCGGAAGTA; ENPP1 *forward* CTATGGACGTGGGGGAGGAG *reverse* TAGGTGTTGGGGTCCTTGGC; and PPARD *forward* GTGGCTTCTGCTCACCAACA *reverse* CATCGTCTGGGTCTGAACGC. The comparative Ct method was used to contrast changes in gene expression levels. β–actin was used as an endogenous control.

### Statistical analyses

For transcriptomic data, statistical analyses were performed using Excel (Microsoft Corporation^®^), statistical significance was determined by student’s *t-* test and false discovery rate (FDR) correction, both of them with α = 0.05. The results were expressed as means ± S.E.M for *n* independent experiments. Statistical significance was determined by two-way ANOVA, with α = 0,05. For transcriptomic data validation, Prism 7 (GraphPad Software) for Mac was used. The results are expressed as means ± S.D. of 3 independent experiments. Two way ANOVA and Tukey’s multiple comparisons test were done^7,26^.

## Electronic supplementary material

Supplementary information tables

Supplementary table 1

Supplementary table 2

Supplementary table 3A

Supplementary table 3B

Supplementary table 4A

Supplementary table 4B

Supplementary table 5A

Supplementary table 5B

Supplementary table 6A

Supplementary table 6B

Supplementary table 6C

Supplementary table 6D

Supplementary table 7A

Supplementary table 7B
