# Supplementary material for: Angiogenesis and evading immune destruction are the main related transcriptomic characteristics to the invasive process of oral tongue cancer
Source: Sci Rep. 2018 Jan 31;8:2007. doi: 10.1038/s41598-017-19010-5 (PMC5792437; doi:10.1038/s41598-017-19010-5)
Supplement: Supplementary file 1 — Supplementary information tables [file 41598_2017_19010_MOESM1_ESM.docx]

**Angiogenesis and evading immune destruction are the main related transcriptomic characteristics to the invasive process of oral tongue cancer.**

Juan Alberto Pérez-Valencia^1^, Francisco Prosdocimi^1^, Italo M. Cesari^1^, Igor Rodrigues da Costa^1^, Carolina Furtado^2^, Michelle Agostini^3^, Franklin David Rumjanek^1,*^.

**Supplementary table 1.** Total amount of partial RNA sequences and its replicates (biological and technical).

**Supplementary table 2.** Differentially expressed genes (DEGs) found between parental and derived cell lines.

**Supplementary table 3A.** Expression ratios between parental and derived cell lines, for *p-*values.

**Supplementary table 3B.** Expression ratios between parental and derived cell lines, for *q-*values.

**Supplementary table 4A.** Protein coding genes (PCG) related to *p-*values’ DEGs.

**Supplementary table 4B.** Protein coding genes (PCG) related to *q*-values’ DEGs.

**Supplementary table 5A.** Non-coding genes (NCG) related to *p-*values’ DEGs.

**Supplementary table 5B.** Non-coding genes (NCG) related to *q-*values’ DEGs.

**Supplementary table 6A**. KEGG pathways and genes related to each hallmark of cancer and each CoGE for *p-* values, based on PCG information, using “Enrichment” and “KEGG pathways” options of STRING.

**Supplementary table 6B.** KEGG pathways and genes related to each hallmark of cancer and each CoGE for *q-* values, based on PCG information, using “Enrichment” and “KEGG pathways” options of STRING.

**Supplementary table 6C**. KEGG pathways and genes related to each hallmark of cancer and each CoGE for the 35.238 annotated genes in the human genome, using “Enrichment” and “KEGG pathways” options of STRING.

**Supplementary table 6D.** KEGG pathways and genes related to each hallmark of cancer and each CoGE for the 3717 DEGs found in our analyses of *q-*values, using “Enrichment” and “KEGG pathways” options of STRING.

**Supplementary table 7A.** Comparison of KEGG pathways and their related DEGs for each CoGE and each hallmark of cancer and invasion and metastasis hallmark, of *p*-values.

**Supplementary table 7B.** Comparison of KEGG pathways and their related DEGs for each CoGE and each hallmark of cancer and invasion and metastasis hallmark, of *q*-values.
